# Depth related adaptations in symbiont bearing benthic foraminifera: New insights from a field experiment on *Operculina ammonoides*

**DOI:** 10.1038/s41598-018-27838-8

**Published:** 2018-06-22

**Authors:** Shai Oron, Sigal Abramovich, Ahuva Almogi-Labin, Julia Woeger, Jonathan Erez

**Affiliations:** 1 Department of Geological and Environmental Sciences, Ben-Gurion University of the Negev, Beer-Sheva, 84105 Israel; 2 The Interuniversity Institute for Marine Sciences (IUI), Eilat, 88103 Israel; 3 The Geological Survey of Israel, Jerusalem, 95501 Israel; 4 Department of Palaeontology, University of Vienna, 1190 Vienna, Austria; 5 The Fredy and Nadine Herrmann Institute of Earth Sciences, The Hebrew University of Jerusalem, Givat-Ram, Jerusalem, 91904 Israel

## Abstract

Large benthic foraminifera (LBF) are marine calcifying protists that commonly harbor algae as symbionts. These organisms are major calcium carbonate producers and important contributors to primary production in the photic zones. Light is one of the main known factors limiting their distribution, and species of this group developed specific mechanisms that allow them to occupy different habitats across the light gradient. *Operculina ammonoides* (Gronovius, 1781) is a planispiral LBF that has two main shell morphotypes, thick involute and flat evolute. Earlier studies suggested morphologic changes with variation in water depth and presumably light. In this study, specimens of the two morphotypes were placed in the laboratory under artificial low light and near the sea floor at depths of 15 m, 30 m, and 45 m in the Gulf of Aqaba-Eilat for 23 days. Differences in growth and symbionts content were evaluated using weight, size, and chlorophyll *a*. Our results show that *O*. *ammonoides* exhibit morphological plasticity when constructing thinner chambers after relocation to low light conditions, and adding more weight per area after relocation to high light conditions. In addition, *O*. *ammonoides* exhibited chlorophyll content adaptation to a certain range of light conditions, and evolute specimens that were acclimatized to very low light did not survive relocation to a high light environment, possibly due to photo-oxidative stress.

## Introduction

Symbiont-bearing larger benthic foraminifera (LBF) generally live in shallow oligotrophic waters of tropical and sub-tropical seas. They mostly harbor endosymbiotic algae and have complicated internal structures^1–3^. They usually exceed 3 mm^3^ in volume^1^, and are an important component of modern low latitudes and ancient oceanic ecosystems. These organisms are major CaCO_3_ producers, but their calcification is a complex physiological process that is biologically controlled and not well understood. Algal symbiosis is prevalent in many LBF species and hence they are considered as major contributors to primary production in the photic zones of shallow seas^4–8^. Studies investigating the biology, ecology, depth distribution, morphology and symbiotic relationship with algae in LBF are essential for monitoring the ecological state of tropical ecosystems, for interpretation of their fossil assemblages and for understanding the impact of future climate and oceanographic changes on marine calcifying organisms.

Symbiont-bearing larger benthic foraminifera distribution in shallow marine environments is determined by a set of parameters, of which water depth has a dominant but indirect role^5,9–13^. Light intensity decreases exponentially with depth because of water absorption and turbidity which are influenced by terrestrial influx and nutrients content^8^. The characteristics of sea water, dissolved materials and suspended particles affect the wave length of the available light in the benthic habitat. It has been suggested that the type of symbiont associated with specific LBF taxa influences foraminiferal depth distribution^14^, and that modifications in the shell shapes may be an adaptation for hosting symbionts^15–17^.

*Operculina ammonoides* (Gronovius, 1781) is a tropical hyaline diatom-bearing LBF, which is found in a wide range of depths^5,16,18–20^. It is closely related to the genus *Nummulites* (within the same sub-family^21^), which were widespread throughout the Paleogene (sub) tropics to the extent that they are the principal component of some shallow-water carbonates^22^. The shell of *O*. *ammonoides* is planispiral and exhibits two main morphotypes with various intermediate forms: thick involute morphotype (Fig. 1.1), in which the chamber outer layers cover laterally those of the preceding coil, and flat evolute morphotype (Fig. 1.2), in which the chamber outer layers in a coil do not laterally cover those of the preceding coil. Quite often intermediate semi-involute morphotypes are also found (Fig. 1.3). Previous studies in the Gulf of Aqaba-Eilat described this species as occupying depth interval of 30–150 m, and being very common at water depths greater than 40 m on sandy substrates^5,16^, with numerical abundance increase of up to 63% in the dead assemblage from 60–90 m^23^. Few studies indicated that specimens found shallower than 40–60 m are mostly more involute and thicker than specimens from deeper water^5,16^.

**Figure 1:**
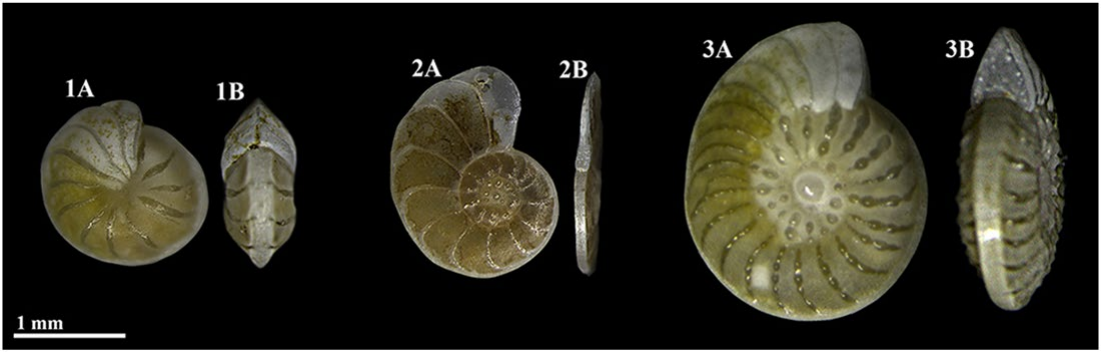
Variability in the mode of coiling in *Operculina ammonoides*. (**1**) Involute. (**2**) Evolute. (**3**) Semi-involute (Advolute/Convolute). (**A**) Side view. (**B**) Apertural view.

Our own observations show that this species also thrives at depths of 15–30 m at the Northern Gulf where it is found on the sandy and silty sediments and around the sea grass *Halophila stipulacea*. In this habitat, it appears in very high densities of 5–10 live individuals per cm^2^, and is a significant component of the dead assemblages^20^.

Previous studies suggest that *O*. *ammonoides* shell changes from involute and semi-involute to evolute with increase in water depth due to lower irradiance and/or current energy^5,9,16,19,24^. The strong linkage between shell morphology and depth is often assigned to irradiance levels that seem to mainly affect the functionality of the diatom symbionts. However, Oron *et al*.^20^ documented variability in the mode of coiling in *O*. *ammonoides* population sampled at 27 m water depth after removal of fish farms from the northern Gulf of Aqaba-Eilat in 2008. This was probably an ecophenotypic response which might be typical to this species in impacted, high turbidity environments, but the origin of this phenomenon and its possible linkage to light levels is unknown.

It was previously demonstrated that changes of morphological characters within *Operculina* and *Planoperculina* species can be used for depth gradient estimation using regression analyses^25^. Such methods can be used for high resolution paleodepth, and possibly water turbidity estimation. Studies of living forms can be combined with the rich nummulitids fossil record to better understand growth history and biostratigraphy implications.

Another depth related hypothesis derives from the fact that some groups of foraminifera exhibit sexual dimorphism. This dimorphism involves an asexual generation with a large proloculus (initial chamber) and small test diameter (megalospheric), and a sexual generation with a small proloculus and large test diameter (microspheric). A possible link to dimorphism was suggested based on an increase in proloculus size with depth in *O*. *ammonoides* from the Red Sea^26^. However, this observation could have been the result of the presence of two types of megalospheric forms, as documented in *Heterostegina depressa* from Hawaii^27^.

In this study, we combined laboratory and unique *in situ* experiments to investigate physiological responses of *O*. *ammonoides* to depth changes. Our results further develop the perception that the actual depth distribution is a compromise between different habitat requirements, and that the foraminifera shell shape must accommodate the needs of the symbionts when living outside their optimum light range. This study also provides important insights on the functional morphology of large benthic foraminifera, and has a direct implication for interpretations of the paleoecology and taxonomy of fossil nummulitids.

## Experimental Design and Methods

### Collection of specimens

For involute specimens, sediments were collected by SCUBA diving from soft bottom sites located at the North Beach of the Gulf of Aqaba-Eilat, at 20 m water depth, where *O*. *ammonoides* is abundant mostly in its involute and slightly semi-involute forms. The sediments were sieved between 1000 µm and 1500 µm and placed in glass beakers with seawater for collection of climbing individuals.

Evolute specimens were collected from culturing containers in the laboratory containing sediments that were brought by technical Closed-Circuit Rebreathing (CCR) diving from 45 m water depth in the coral reef area off the Inter-University Institute for Marine Sciences in Eilat (IUI). The evolute specimens that were collected for this study were part of a newly formed gamont generation, produced by asexual reproduction of the original population. The laboratory cultures were maintained under low light conditions of ~10 μmol photons m^−2^ s^−1^ supplied by a white Light-Emitting Diode (LED) lamp and at 24–25 °C, the average annual temperature in the northern Gulf of Aqaba-Eilat.

### Experimental setup

After the collection of live individuals, 110 involute (and slightly semi-involute) and 55 evolute adult specimens were divided into 11 groups of 15 specimens (10 involute, and 5 evolute) and cleaned under the binocular microscope using brushes. All specimens were photographed, and two groups were frozen for later analysis. The nine groups intended for translocation were labeled with the fluorescent probe calcein to indicate the addition of new chambers by cultivating them in filtered seawater with calcein 20 μM for two days. The experimental groups were placed in transparent, perforated (~1 mm) 10 mL plastic tubes. The tubes were attached to small weights and placed near the sea floor at depths of 15 m (three groups), 30 m (three groups) and 45 m (two groups) off the coast of the IUI marine laboratory. One group (“80” m) was placed in the laboratory under low light conditions (~10 μmol photons m^−2^ s^−1^) approximating the maximum light levels at ~80 m off the marine laboratory, and was cultured inside the same plastic tubes as the *in situ* groups in a seawater aquarium in 24–25 °C. The water in the aquarium was changed every 4–5 days with fresh unfiltered sea water collected off the IUI marine laboratory pier. All experimental tubes were cleaned after 11 days, and retrieved after 23 days.

Additional 10 thick involute specimens, that were left after the assembly of the experiment groups, were calcein labeled for 6 days and cultivated in the low light environment of the laboratory for a longer period (43 days) for growth observation purposes.

In addition, 38 involute and 39 evolute dead specimens (empty shells) were collected from the same sediments and culturing containers as the live individuals for establishing a weight to area relationship chart on representative “standard shaped” specimens of both morphotypes.

### Temperature and light at the study sites

**Figure 2:**
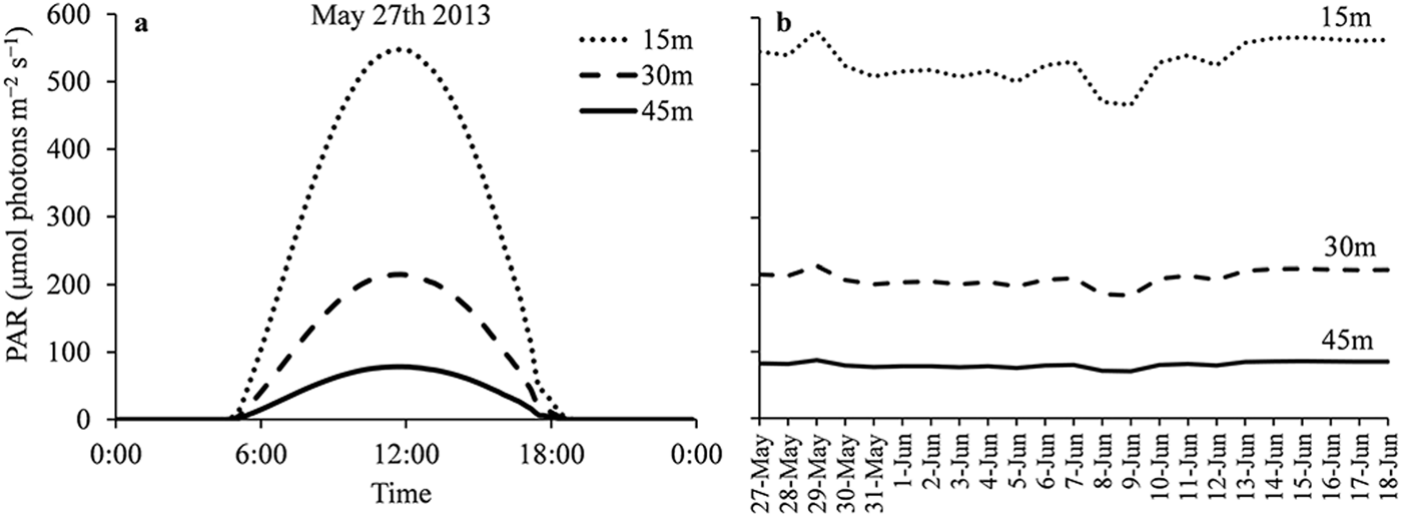
Typical daily light profile (**a**) and maximum light levels (**b**) during the experiment for the *in situ* sites at depths of 15 m, 30 m and 45 m, off the IUI Marine Laboratory (May-June 2013). PAR = Photosynthetically Active Radiation.

**Figure 3:**
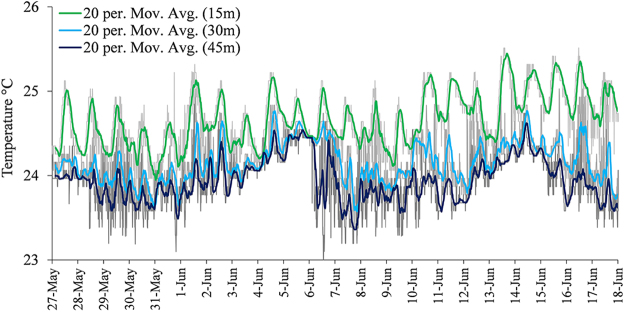
Temperature fluctuations throughout the *in situ* experiment at depths of 15 m, 30 m and 45 m in the experimental sites off the IUI Marine Laboratory (May-June 2013). Color lines are 20 period moving average.

During the experiment (May–June 2013), HOBO^®^ Pendant Temperature/Light Data loggers were attached to the plastic tubes recording light levels in Lux units (lumen m^−2^) and temperature. Logging was done at 10 min intervals throughout the experiment. Photosynthetically Active Radiation (PAR), in units of μmol photons m^−2^ s^−1^, was calculated using available surface PAR data logged at the meteorological station on the marine laboratory pier (http://www.iui-eilat.ac.il/Research/NMPMeteoData.aspx), combined with typical light attenuation for the month of May in the coral reef area off the IUI marine laboratory^28^. The maximum daily PAR calculated for depths of 15 m, 30 m and 45 m was 566 μmol photons m^−2^ s^−1^, 222 μmol photons m^−2^ s^−1^ and 81 μmol photons m^−2^ s^−1^ respectively (Fig. 2). *In situ* temperatures fluctuated between 23.1 °C and 25.5 °C at 15 m, 23.3 °C and 25.1 °C at 30 m and 23 °C and 24.8 °C at 45 m (Fig. 3). The collection site of the involute specimens in the North Beach is characterized by higher turbidity, and the seasonal maximum light levels in 20 m are ~180 μmol photons m^−2^ s^−1^, a lower light level than the same depth off the marine laboratory in the south beach (Tamir, unpublished data). However, the involute specimens that were collected there spent ~5 days in the low light environment of the laboratory before the beginning of the experiment.

### Analytical techniques

#### Digital and x-ray imaging

Digital images of all specimens were taken before and after the experiment for measurements of shell growth. Normal light photography was done using a Leica stereomicroscope with fitting digital camera. The photographs after the experiment were taken under very low light using high camera sensor sensitivity (ISO) function, to minimally affect the symbionts. Epifluorescent photography was done using a Leica stereomicroscope with a Leica DFC310 FX camera. X-ray computed microtomography on 10 representative specimens was done with Skyscan 1173 Desktop-Micro-CT scanner (Bruker) at the Institute of Paleontology, University of Vienna.

#### Size/weight measurements and growth calculations

Area measurements were done with Photoshop analysis measurement tools. Area increase was calculated for each individual using the start and end overhead view area.

Weight increase was estimated for each individual using the start calculated weight based on measured area and the weight vs area charts established from empty shells, and the end weight measured after the experiment. Broken specimens were excluded. At the end of the experiment it became clear that the two days cultivation in calcein was not sufficient to label all individuals, so all growth calculations were done using the methods mentioned above.

#### Chlorophyll measurements

At the end of the experimental period, specimens were frozen in −18 °C and stored in the dark until analyzed for chlorophyll *a*. Randomly selected specimens from each group were placed in a glass vial containing 2 mL of acetone (90%) in 4 °C in the dark. After 24 h, the extracted chlorophyll was measured using a Chl *a* Acidification Fluorescent Module in a Turner Designs Trilogy Laboratory Fluorimeter by following EPA Method 445.0^29^. Most measurements were done on a single specimen, to inspect the variability within the population. Specimens used for chlorophyll analysis were later treated in 5% sodium hypochlorite (NaOCl) for 12 h for removing organic matter, and the remaining calcium carbonate was weight to normalize chlorophyll values.

### Statistical analysis

Most datasets were found to have non-normal distribution after using Shapiro-Wilk test and histograms observations (Supplementary Table S1), so the Mann-Whitney nonparametric test was used for comparing means. Shapiro-Wilk test and Mann-Whitney test were performed with IBM SPSS Statistics 22 (IBM Corp., Armonk, USA).

### Data availability

All data generated or analyzed during this study are included in this article and its Supplementary Information files.

## Results

### Growth and morphology

**Figure 4:**
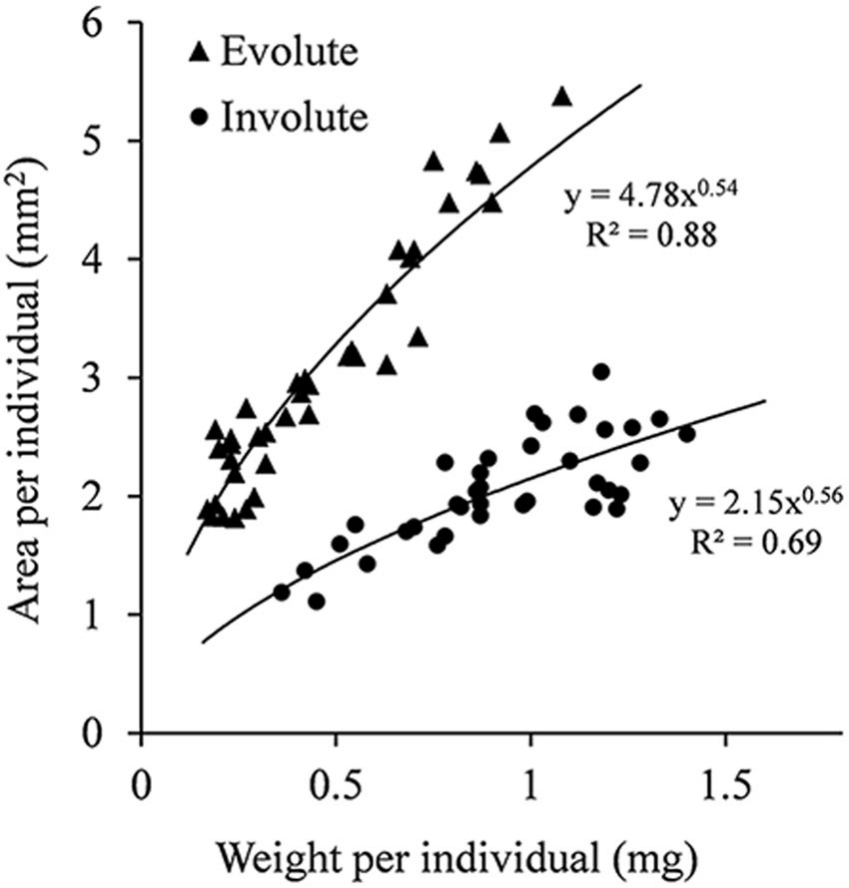
Area and weight relationships in adult involute and evolute specimens of *O*. *ammonoides*. Based on measurements of 38 involute and 39 evolute dead specimens (empty shells) collected from the same sediments and culturing containers as the live individuals used in this experiment.

**Figure 5:**
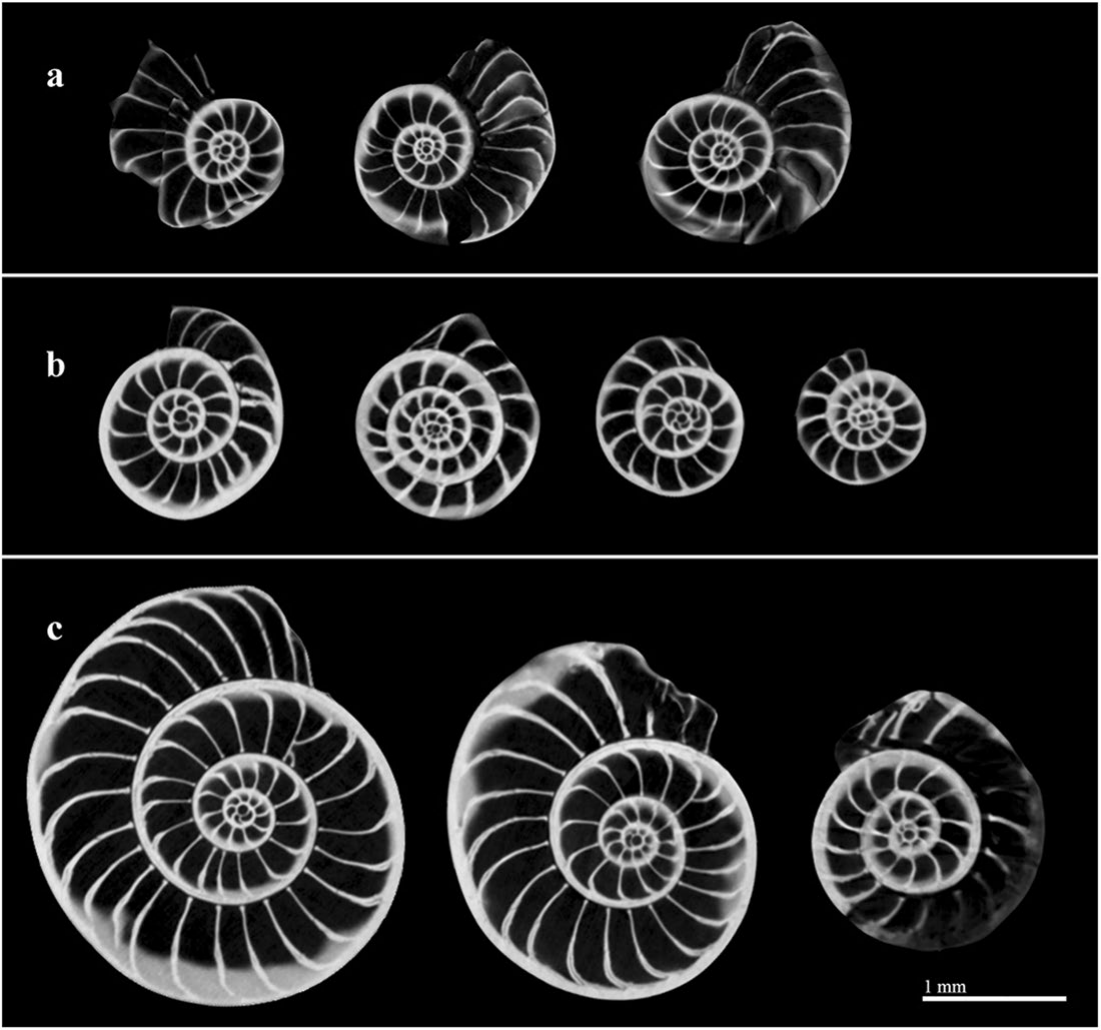
Computed Tomography scan images of evolute (**a**), involute (**b**) and semi-involute (**c**) *O*. *ammonoides* specimens all showing megalospheric proloculus (large and visible initial chamber). The evolute specimens are from the laboratory and the involute specimens were collected in the field (North Beach 15–20 m).

The two main morphotypes have a distinctively different weight-to-area relationship (Fig. 4). Namely, envolute specimens are distinctly larger in area per unit of weight (by a factor of ~2). Computed Tomography (CT) scan images of selected specimens of different morphologies from the collection sites all showed megalospheric proloculus with average diameter of 59(±14) µm (Fig. 5), which indicates that the origin of those specimens is by asexual reproduction.

**Figure 6:**
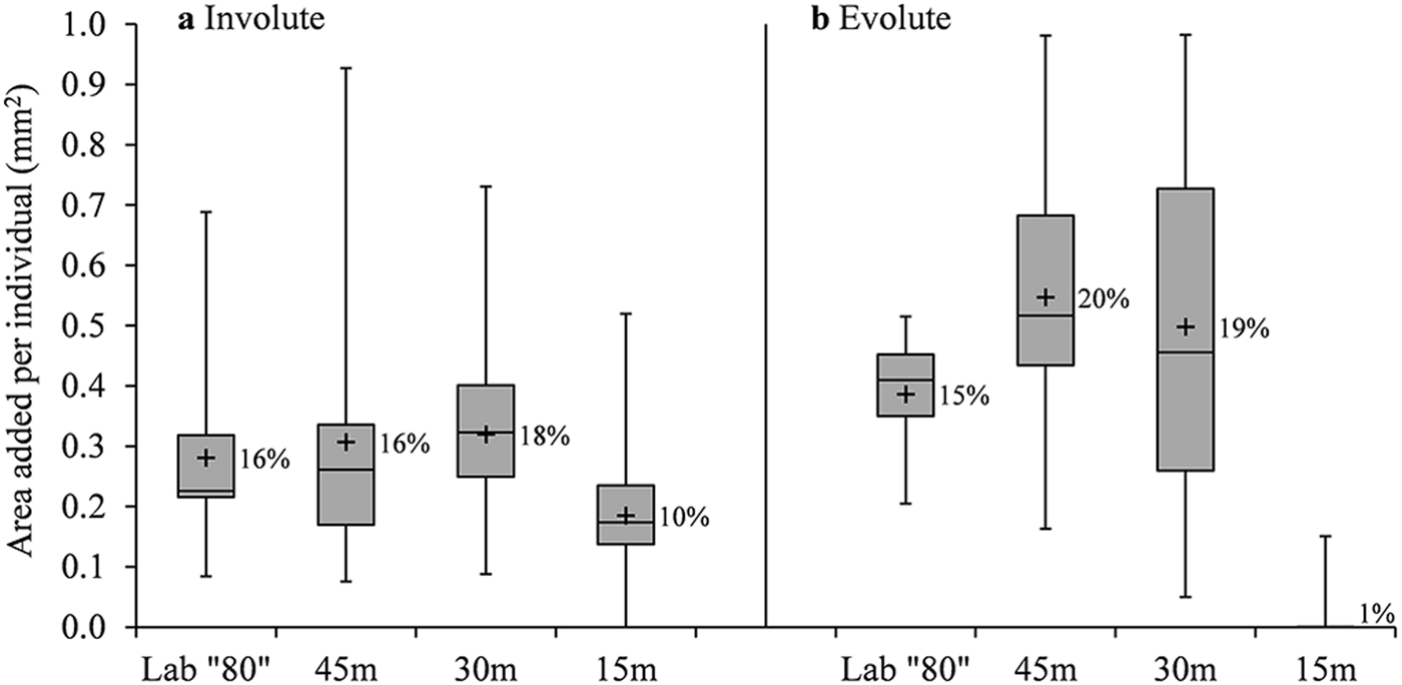
Area increase per individual in “80” m, 45 m, 30 m and 15 m after 23 days of *in situ* deployment on the sea floor. In both Involute (**a)** and Evolute (**b)** specimens, the difference between the 15 m group and the other groups is statistically significant (p < 0.050, Supplementary Table S2). Number of analyzed specimens (n) from left to right for Involute: 10, 20, 30, 30 and Evolute: 5, 10, 15, 15. Box-plot elements: whiskers = most extreme values, middle line = median, upper and lower lines = quartiles, +  = Average. Average area increase in percentage per individual is presented next to the box.

Growth in both morphotypes showed high variability between individuals. Area increase for involutes was statistically lower in 15 m (p < 0.050, Supplementary Table S2), where average growth per individual was 10%(±5%) compared with 17%(±9%) in the deeper groups (Fig. 6a). All evolute specimens from 15 m were bleached and dead upon retrieval (no pseudopodia activity and no color), and growth in 30 m, 45 m and “80” m was statistically similar (Supplementary Table S2) with an average of 19%(±10%) per individual (Fig. 6b).

**Figure 7:**
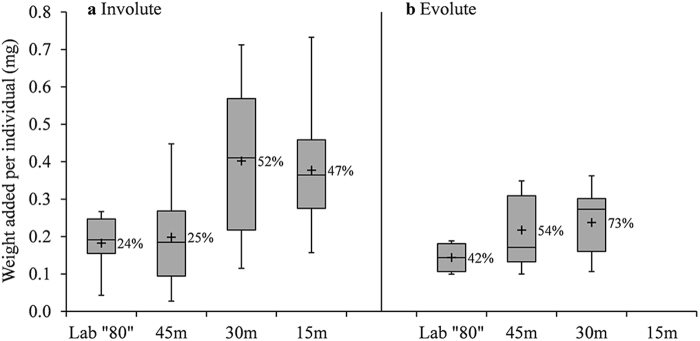
Estimated weight increase per individual in “80” m, 45 m, 30 m and 15 m after 23 days of *in situ* deployment on the sea floor. In Involute (**a**) specimens the difference between the high and intermediate light groups (15 m and 30 m) and the lower light groups (45 and “80” m) is statistically significant (p < 0.002, Supplementary Table S3). In Evolute (**b)** specimens the 15 m group did not show detectible weight increase, and all other groups are statistically similar (Supplementary Table S3). Number of analyzed specimens (n) from left to right for Involute: 10, 18, 30, 25 and Evolute: 4, 9, 14, 12. Box-plot elements: whiskers = most extreme values, middle line = median, upper and lower lines = quartiles, +  = Average. Average area increase in percentage per individual is presented next to the box.

Estimated weight addition for involutes was statistically higher in 15 m and 30 m compared with 45 m and “80” m (p < 0.002, Supplementary Table S3), with an average of 24%(±16%) per individual in the shallower groups and 50%(±23%) per individual in the deeper groups (Fig. 7a). Estimated weight addition for evolutes was statistically similar (Supplementary Table S3) for all surviving groups, with an average of 62%(±27%) per individual (Fig. 7b).

**Figure 8:**
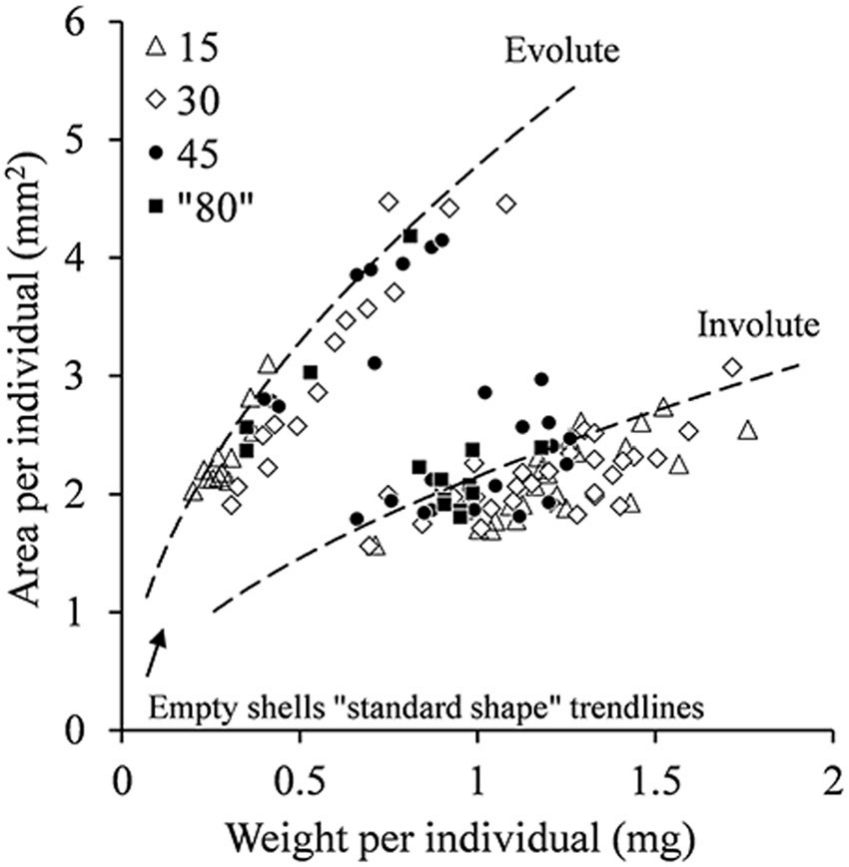
Area vs. weight of all individuals after the experiment and the “standard shape” trendlines from Fig. 4. Most individuals from 15 m and 30 m are heavier compared to the control population of the same area.

When comparing area vs weight of the individuals after the experiment with the “standard shape” trendlines from Fig. 4, most individuals from 15 m and 30 m appear under the trendline, meaning they are heavier compared to the “standard shape” population of the same area or diameter (Fig. 8).

**Figure 9:**
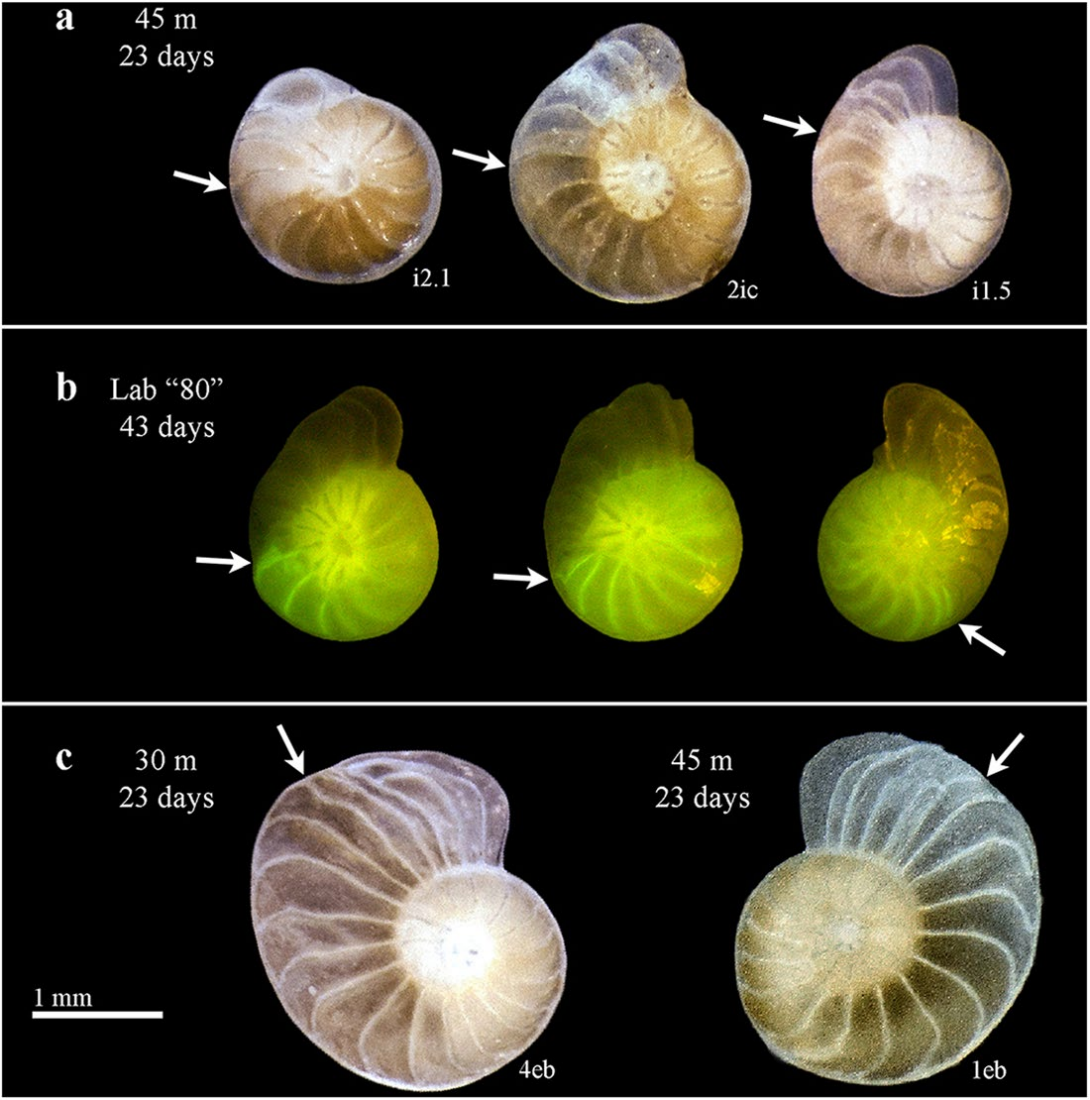
Growth and morphology changes during the experiment. Involute specimens with “evolute-like” thinner chambers after relocation to 45 m for 23 days (**a**), and to “80” m (laboratory) for 43 days (**b**, calcein-labeled), and irregular chamber formation in evolute forms (**c**). The arrows indicate the beginning of the experiment.

Involute specimens that formed more than ~3 new chambers in the deep *in situ* groups, visibly show thinner chambers which are more consistent in shape and coiling mode with evolute forms (Fig. 9a). The latter observation is even more visible in the additional 10 thick involute specimens that were cultivated in the “80” m conditions for 43 days (Fig. 9b). Abnormal chamber formation was also observed, mostly in evolute specimens, forming chambers with shorter septal distance, shorter chamber height or longer backbend angle (Fig. 9c).

### Chlorophyll

**Figure 10:**
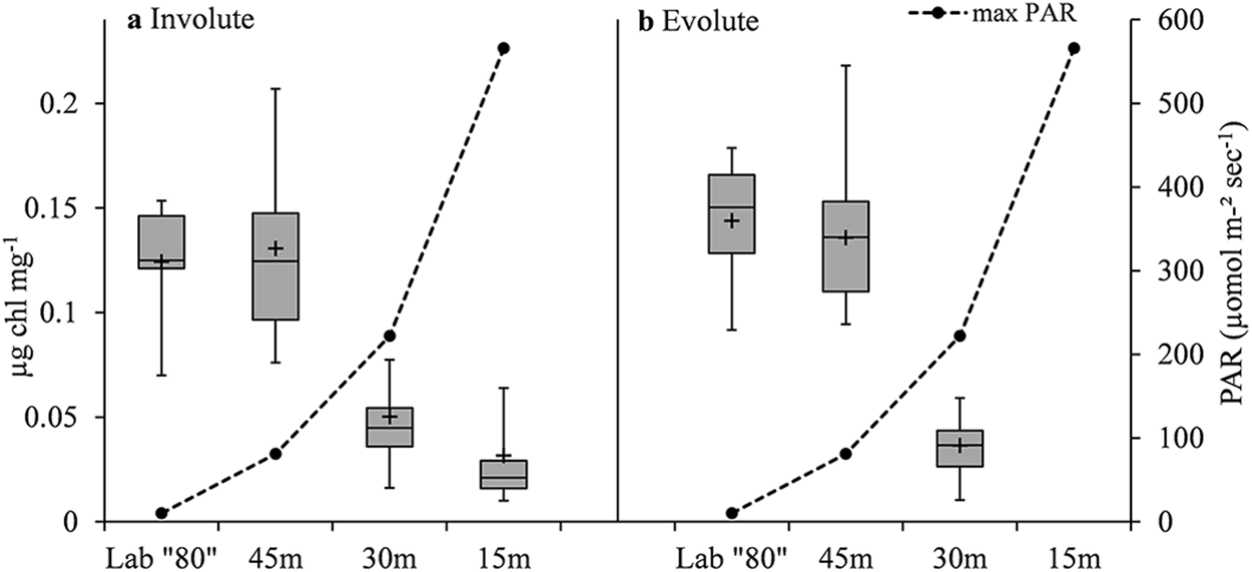
Chlorophyll *a* content per mg in “80” m, 45 m, 30 m and 15 m. In both involute (**a**) and evolute (**b)** specimens the difference between the low light conditions (“80” m and 45 m) and the intermediate/high light conditions (30 m and 15 m for involute and 30 m for evolute) is statistically significant (p < 0.002, Supplementary Table S4). All evolute specimens at 15 m were bleached and dead upon retrieval. Number of analyzed specimens (n) from left to right for Involute: 10, 8, 8, 8 and Evolute: 6, 8, 8, 8. Box-plot elements: whiskers = most extreme values, middle line = median, upper and lower lines = quartiles, + = Average. PAR = Photosynthetically Active Radiation.

In both involute and evolute morphotypes chlorophyll *a* content in “80” m and 45 m was statistically higher (by a factor of ~3–4) than in 30 m and 15 m (p < 0.002, Supplementary Table S4), excluding the evolute individuals from 15 m that were bleached and dead. Average Chlorophyll *a* values for involutes were 0.13(±0.03) µg mg^−1^ in the deeper groups and 0.04(±0.02) µg mg^−1^ in the shallower groups (Fig. 10a), and average Chlorophyll *a* values for evolutes was 0.14±0.03 µg mg^−1^ in the deeper groups and 0.03±0.02 µg mg^−1^ in the 30 m group (Fig. 10b). Chlorophyll *a* content negatively correlates to average maximum daily light at the different depths (Fig. 10).

## Discussion

Interactions between environmental factors often make it difficult to correlate ecomorphology of LBF with depth distribution and other variables. Haynes^15^ suggested that shell shape is a compromise between available light, metabolic requirements associated with algal symbiosis and hydrodynamic factors. Many early studies have documented LBF morphology changes with variation in habitat depth, light levels and water motion, and the authors mostly attributed the changes to the association with symbionts. Specifically, for some key species of diatom-bearing LBF (*Amphistegina*, *Heterostegina* and *Operculina*), it has been shown that thicker tests are found in shallow water^5,6,19,30–33^ and when cultivated under high light levels^32,34^. In addition, *Amphistegina* spp. are thicker in habitats that are exposed to wave action, and produce thicker shells when subjected to water motion during growth in the laboratory^32,34,35^. Sectioned tests revealed that this feature is a direct result of differences in secondary lamellar thicknesses^33,34^.

For *O*. *ammonoides* specifically, where involute specimens are distinctively heavier compared to evolute specimens of the same size (Fig. 4), the thickening of the test in shallow water appear to be related to changes in the mode of coiling, and the lateral surface of the chamber walls is greater in deeper water^5,19,30^. It was also concluded that all *O*. *ammonoides* ecotypes belong to the same species based on biometric measurements of specimens from different depths, and megalospheric forms were observed among involute and evolute specimens^5^. Computed Tomography scan images of selected specimens of different morphologies from our collections all showed megalospheric forms (Fig. 5). Therefore, the morphological variability is not related to the sexual or asexual origin of the individuals.

As for growth rates in diatom-bearing LBF, previous work on *H*. *depressa* showed optimum growth rates in very low light levels of 300 “daylight neon” Lux (~5 μmol photons m^−2^ s^−1^)^36,37^, and in 600 Lux (~10 μmol photons m^−2^ s^−1^)^38^. Erez^39^ described a mid-water (25 m) growth and photosynthesis optimum in the diatom-bearing *A*. *lobifera* based on *in situ* experiments, and excluded the simple notion that growth rates decrease with depth as a result of reduction in photosynthetic activity. Furthermore, the 25 m optimum contradicts the reports describing the preferred habitat of *A*. *lobifera* (<10 m), implying that the actual depth distribution is a compromise between different habitat requirements, and that the foraminiferal test shape must accommodate the needs of the symbionts when living outside their optimum light range.

In this study, *O*. *ammonoides* translocated thick involute specimens showed higher area growth in the lower light conditions of the “80” m, 45 m and 30 m compared to 15 m (Fig. 6a), but higher weight addition in 15 m and 30 m (Fig. 7a). The temperature in the laboratory (24–25 °C) was slightly higher and more stable than in the deep *in situ* sites (Fig. 3), there was no continuous water motion and the irradiance was constant. However, those parameters did not have a significant effect on survival and growth. Therefore, it is reasonable to assume that the main parameter favoring area growth in this experiment was relatively low light levels of a wide range (~10–222 μmol photons m^−2^ s^−1^).

Specimens exhibited morphological plasticity when individuals from 15 m and 30 m became heavier compared to the control population of the same size (Fig. 8), and involute specimens that were relocated to the low light level environments formed new thinner chambers, which were consistent in shape with the evolute forms (Fig. 9a,b). The latter observation agrees with the light-controlled morphology hypothesis described for *O*. *ammonoides*, and also indicates that individuals can adapt to new conditions, and balance the thickness and surface area of the test depending on the incoming light.

Another observation we report here is abnormal growth of many of the newly formed chambers during the experiment. The abnormalities include deformed smaller chambers and chambers with shorter septal distance, shorter chamber height or longer chamber backbend angle. The abnormalities are mostly visible in the evolute forms from the *in situ* groups (Fig. 9c), but appear to some extant in all experiment groups, including the laboratory grown ones (Supplementary Figs S1–S9). Previous explanations for deformity in foraminifera include mostly natural environmental stressors and pollution^40–42^ or regeneration after damage^43^. However, in our experiment, we cannot account for those variables. The common parameter for almost all groups is the relocation and abrupted change in conditions, that may have caused disruption of the cytoskeleton.

Corals that are adapted to low light and were relocated to shallow water usually show high mortality rates^44–47^ and/or bleaching^48,49^. Similarly, LBF have suffered documented bleaching incidents in recent decades. Cases of LBF field populations bleaching are documented in four species of the diatom-bearing *Amphistegina* (reviewed by Hallock *et al*.^50^). Negative response of LBF to high light levels in experiments in the laboratory and *in situ* was also documented in some cases: *A*. *lobifera*, a shallow dwelling (>10 m) species, showed a mid-water (25 m) optimum for growth and photosynthesis^39^ when cultivated in jars *in situ* and unable to interact with the substrate (i.e. cryptic behavior). In the laboratory, light levels above *A*. *lobifera* optimal range induced oxidative stress and bleaching and caused significant reduction in survivorship^51^. *Amphistegina lessonii*, typically a deeper-dwelling species (20–40 m), was photoinhibited in full sunlight. *Heterostegina depressa*, a diatom-bearing Nummulitidae like *O*. *ammonoides*, has reduced growth rates and maximum quantum yields in a very high light regime (with midday peaks at 1200 μmol photons m^−2^ s^−1^)^52^, and showed signs of stress even under illumination of 100 μmol photons m^−2^ s^−1 ^^53^. A key difference between the known cases of bleaching in corals and bleaching in LBF is that events of bleaching in corals are mostly correlated with elevated temperatures^45^, while bleaching in LBF is associated with photoinhibitory stress^54–56^. However, temperature-induced bleaching was reported in three species of diatom-bearing LBF^57^, and it was shown that more specimens of *Amphistegina gibbosa* were partially bleached under high light intensities in 32 °C compared with 20 °C and 25 °C^55^. A current prevailing model for bleaching propose a primary trigger of light-dependent generation of reactive oxygen species by heat-damaged chloroplasts^58,59^, therefore while bleaching in LBF is linked mostly to light levels, other factors such as temperature, or a combination of factors, may also induce bleaching.

In this study, the evolute specimens that were adapted to low light in the laboratory did not survive the relocation to 15 m (max light levels of 566 μmol photons m^−2^ s^−1^). All other groups in this experiment at all depths showed 100% survival. The low-light adapted evolute specimens possibly experienced photo-oxidative stress, as described in corals and in some species of foraminifera. Unlike the relocated evolute specimens, the involute ones could sustain the higher light level at 15 m probably due to their thinker morphology that is more protective of the symbionts since they were collected in the higher (compared to the laboratory) light environment of the North Beach.

Diatom-bearing LBF are a diverse group, and the diversity of diatoms in symbiosis with foraminifera is comparably high. This diversity may be the reason for the wide ecological range of depth and light habitats of this group and their high abundances in those habitats^52^, and photosynthetic plasticity may be the tribute that allows some species to acclimatize rapidly to different light conditions^53^. Over the years, *O*. *ammonoides* have been suggested to host various species of diatoms belonging to the genera *Amphore*, *Achnanthes*, *Nitzschia*^60,61^ and *Thalassionema*^62^. It has long been known that diatoms can survive at very low light levels and can tolerate relatively high light levels for limited periods of time^63^. Furthermore, improved growth under low irradiances (<11 μmol photons m^−2^ s^−1^) was reported for many diatom species^64–66^, including species that were found in large quantities in *O*. *ammonoides*^60^. The above-mentioned experiments used free living or isolated diatoms, and it should be emphasized here that our study highlights the fact that the hosts test also play a role in controlling and modulating the light received by the endosymbiotic algae. The structure and test shape of the host may have evolved in response to the requirements of the symbionts, and the test shape and thickness balance the surface to volume ratios depending on the incoming light^31,33,67^. In addition, our study shows that light quantity and quality adaptation mechanism in the holobiont level can be also achieved by increasing the biomass and number of symbionts, or by increasing cellular chlorophyll *a* content of the symbionts (Fig. 10), as documented in corals and their zooxanthellae^68–73^.

Interestingly, although growth rates did not differ significantly between the low light environments of the laboratory (approximating maximum light at ~80 m), 30 m and 45 m (Fig. 6), the chlorophyll content adaptation seems to have a threshold somewhere between 30 m and 45 m. Both ecotypes show similar chlorophyll content in the “80” m and 45 m, and much lower values in 30 m (Fig. 10). This phenomenon cannot be explained solely by the exponential nature of light attenuation, and might be related to the different components of the light spectrum. The laboratory artificial light, approximating max PAR at ~80 m, was provided by a cold white LED lamp. Cold white LED typically peaks at ~450 nm (blue light), which is similar to the light components reaching the water column below ~30 m in our *in situ* site, where no light above 600 nm reach 40 m, creating an irradiance field dominated by blue light^74^. During the last few decades researchers documented specific effects of different light components on the photosynthetic rates and pigment content of various species of marine algae. It is known that the spectral composition of light plays a crucial role in growth rate, photoprotective mechanisms and pigment content in diatoms^75^. Some species of diatoms grown in blue and blue-green light had distinctively more chlorophyll compared to full spectrum white light of the same intensity.

As for the holobiont level, in the LBF *A*. *gibbosa*, higher-energy blue light induced more bleaching than lower-energy white light, but growth rates (in diameter) were higher under blue light. The higher growth rates were explained by the exposure to 20% more useable energy^56^. Calcification in high light (full spectrum) adapted corals was mostly enhanced after transfer to blue light, but photosynthesis was observed to be significantly less efficient^74,76,77^. It was suggested that the corals from shallow water are adapted to full light spectra whereas deep corals are adapted to blue light spectra which require more pigments, and that blue light photoreceptors in the coral tissue might be the link between light absorption by the coral host and activation of biological processes that enhance calcification^77^. Similar mechanisms probably exist in LBF, and chromatic adaptation provide selective advantages by maximizing photosynthetic activity under different spectral conditions.

Finally, it should be noted that light enhanced calcification (LEC) was shown to be independent of symbiont photosynthesis both in planktonic and benthic foraminifera using a photosynthetic inhibitor^78,79^. More recently it was shown that LEC in corals could proceed almost without photosynthesis of the symbionts under dark blue light^77^. The implications of these observations for *O*. *ammonoides* will need to be evaluated in future work.

## Electronic supplementary material

Dataset 1

Supplementary information
