# Author Correction: Depth related adaptations in symbiont bearing benthic foraminifera: New insights from a field experiment on *Operculina ammonoides*

**DOI:** 10.1038/s41598-018-29216-w

**Published:** 2018-07-19

**Authors:** Shai Oron, Sigal Abramovich, Ahuva Almogi-Labin, Julia Woeger, Jonathan Erez

**Affiliations:** 1 Department of Geological and Environmental Sciences, Ben-Gurion University of the Negev, Beer-Sheva, 84105 Israel; 2 The Interuniversity Institute for Marine Sciences (IUI), Eilat, 88103 Israel; 3 The Geological Survey of Israel, Jerusalem, 95501 Israel; 4 Department of Palaeontology, University of Vienna, 1190 Vienna, Austria; 5 The Fredy and Nadine Herrmann Institute of Earth Sciences, The Hebrew University of Jerusalem, Givat-Ram, Jerusalem 91904 Israel

Correction to: *Scientific Reports* 10.1038/s41598-018-27838-8, published online 22 June 2018

The Acknowledgements section in this Article is incomplete.

“We wish to express our gratitude to the Inter-University Institute (IUI) of Eilat for providing the facilities to carry out this study. We thank Gal Eyal for accompanying Shai Oron on her CCR diving sessions. The research was supported by the Israel Science Foundation grant No 587/2013. This research is a part of the first author’s PhD dissertation, and was also funded by the Department of Geological and Environmental Sciences at Ben-Gurion University of the Negev and by the Inter-University Institute of Marine Sciences in Eilat (IUI).”

should read:

“We wish to express our gratitude to the Inter-University Institute (IUI) of Eilat for providing the facilities to carry out this study. We thank Gal Eyal for accompanying Shai Oron on her CCR diving sessions. The research was supported by the Israel Science Foundation grant No 587/2013. Part of this work was funded by the Austrian Science Fund Project P26344-B25 ‘Breakthroughs in Growth Studies on Larger Benthic Foraminifera’ the scans were performed using the MicroCT Facility of the Department of Palaeontology at the University of Vienna, Austria. This research is a part of the first author’s PhD dissertation, and was also funded by the Department of Geological and Environmental Sciences at Ben-Gurion University of the Negev and by the Inter-University Institute of Marine Sciences in Eilat (IUI).”

In addition, the legend of Figure 9 is incorrect:

“Growth and morphology changes during the experiment. Involute specimens with “evolute-like” thinner chambers after relocation to 45 m for 23 days (**a**), and to “80” m (laboratory) for 43 days (**b**, calcein-labeled), and irregular chamber formation in evolute forms (**c**). The arrows indicate the beginning of the experiment.”

should read:

“Growth and morphology changes during the experiment. Involute and slightly semi-involute specimens with “evolute-like” thinner chambers after relocation to 45 m for 23 days (**a**), and to “80” m (laboratory) for 43 days (**b**, calcein-labeled), and irregular chamber formation in evolute forms (**c**). The arrows indicate the beginning of the experiment.”

