# Supplementary material for: Depth related adaptations in symbiont bearing benthic foraminifera: New insights from a field experiment on Operculina ammonoides
Source: Sci Rep. 2018 Jun 22;8:9560. doi: 10.1038/s41598-018-27838-8 (PMC6015019; doi:10.1038/s41598-018-27838-8)
Supplement: Supplementary file 2 — Supplementary information [file 41598_2018_27838_MOESM2_ESM.pdf]

Supplementary information

Depth related adaptations in symbiont bearing benthic foraminifera: New insights from a field experiment on *Operculina ammonoides*

Shai Oron[1,2*], Sigal Abramovich[1,2], Ahuva Almogi-Labin[3], Julia Woeger[4], Jonathan Erez[5]

[1]Department of Geological and Environmental Sciences, Ben-Gurion University of the Negev, Beer-Sheva, Israel

[2]The Interuniversity Institute for Marine Sciences (IUI), Eilat, Israel

[3]The Geological Survey of Israel, Jerusalem, Israel

[4]Department of Palaeontology, University of Vienna, Vienna, Austria

[5]Earth Science Institute, the Hebrew University, Jerusalem, Israel

***Corresponding author email: shaioro@post.bgu.ac.il.**

Supplementary figures

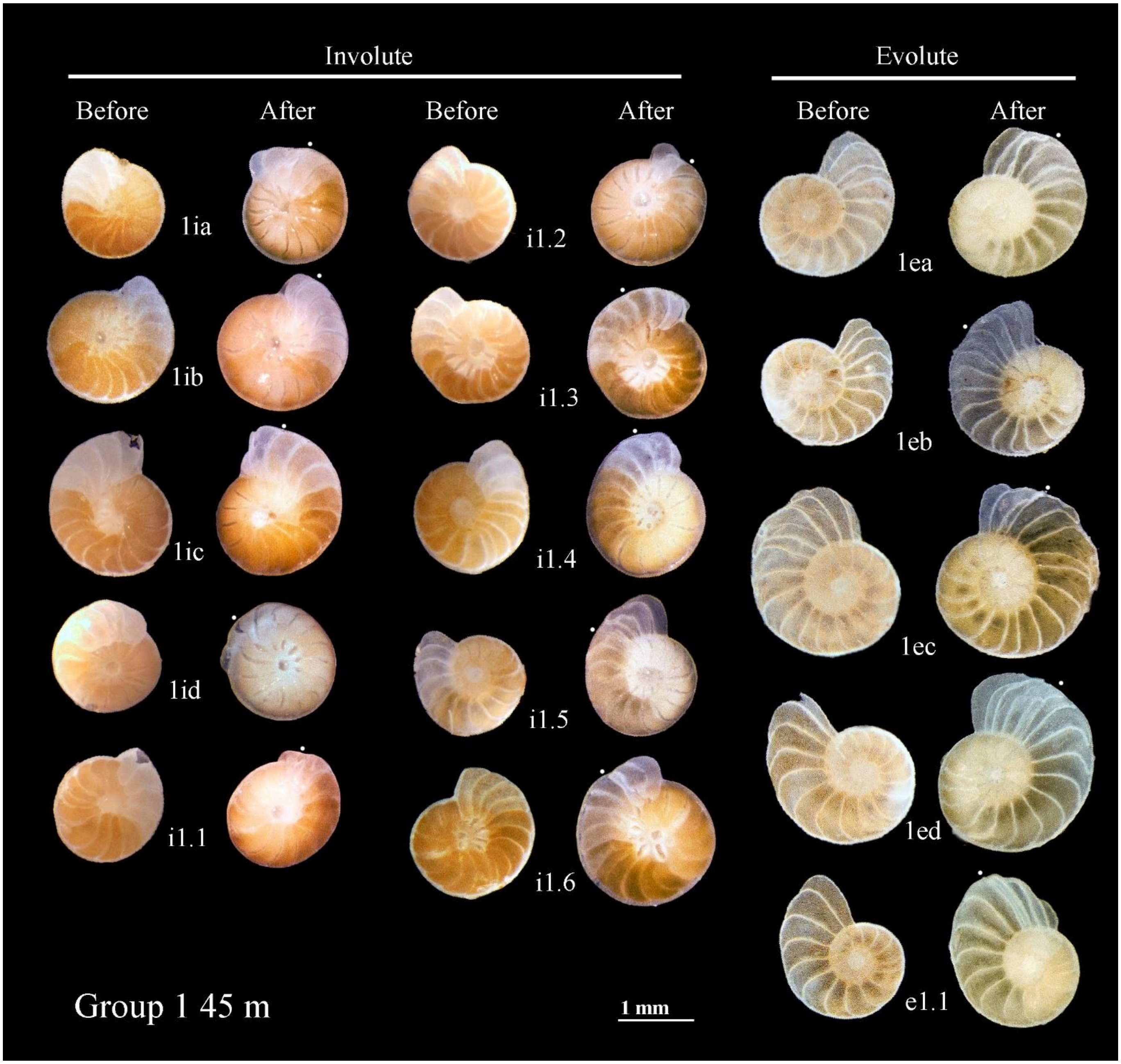

Figure S1: Photographs of all specimens from group 1 before and after the experiment. Labels represent specimen ID. White dots mark the newly formed chambers.

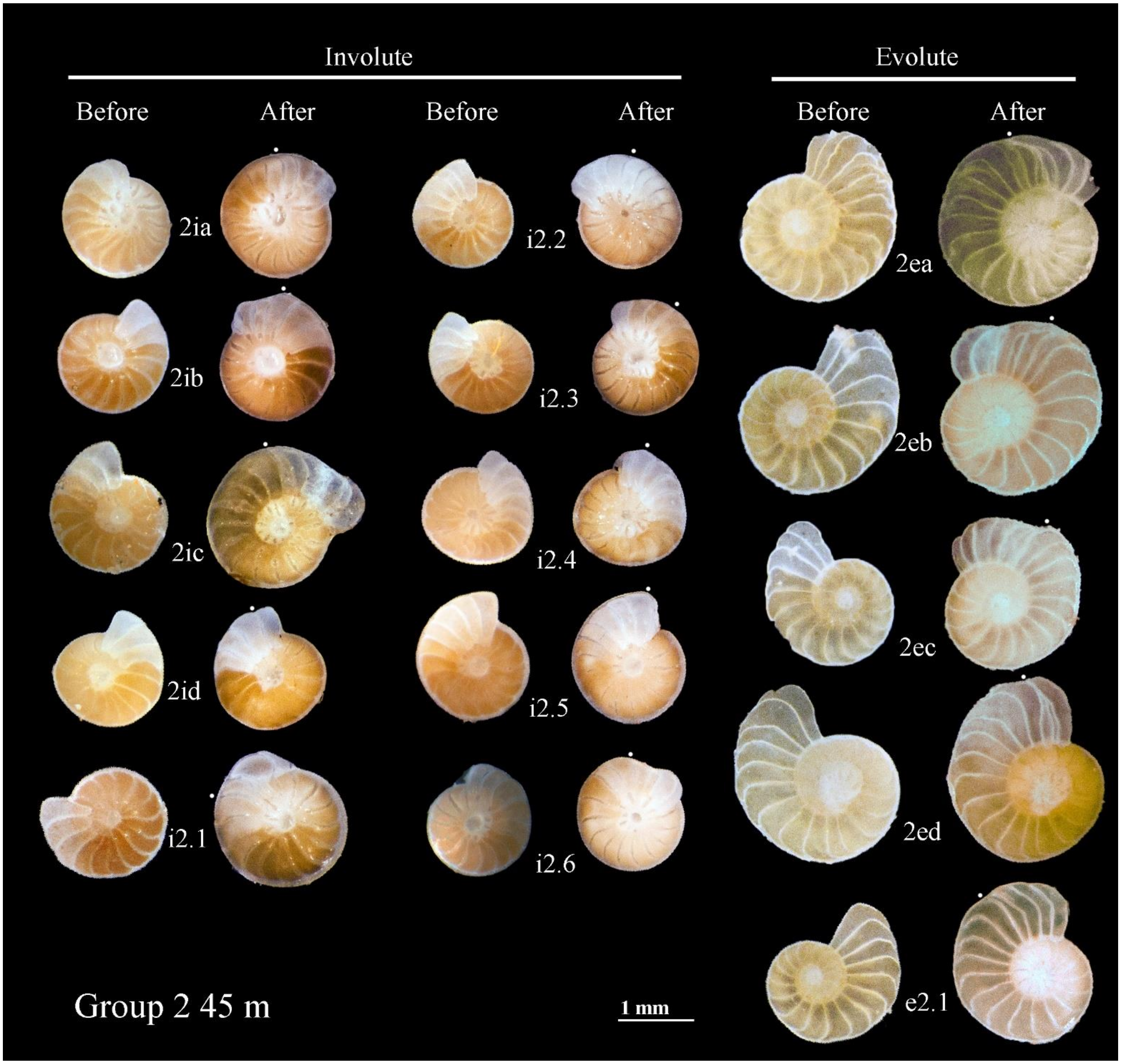

Figure S2: Photographs of all specimens from group 2 before and after the experiment. Labels represent specimen ID. White dots mark the newly formed chambers.

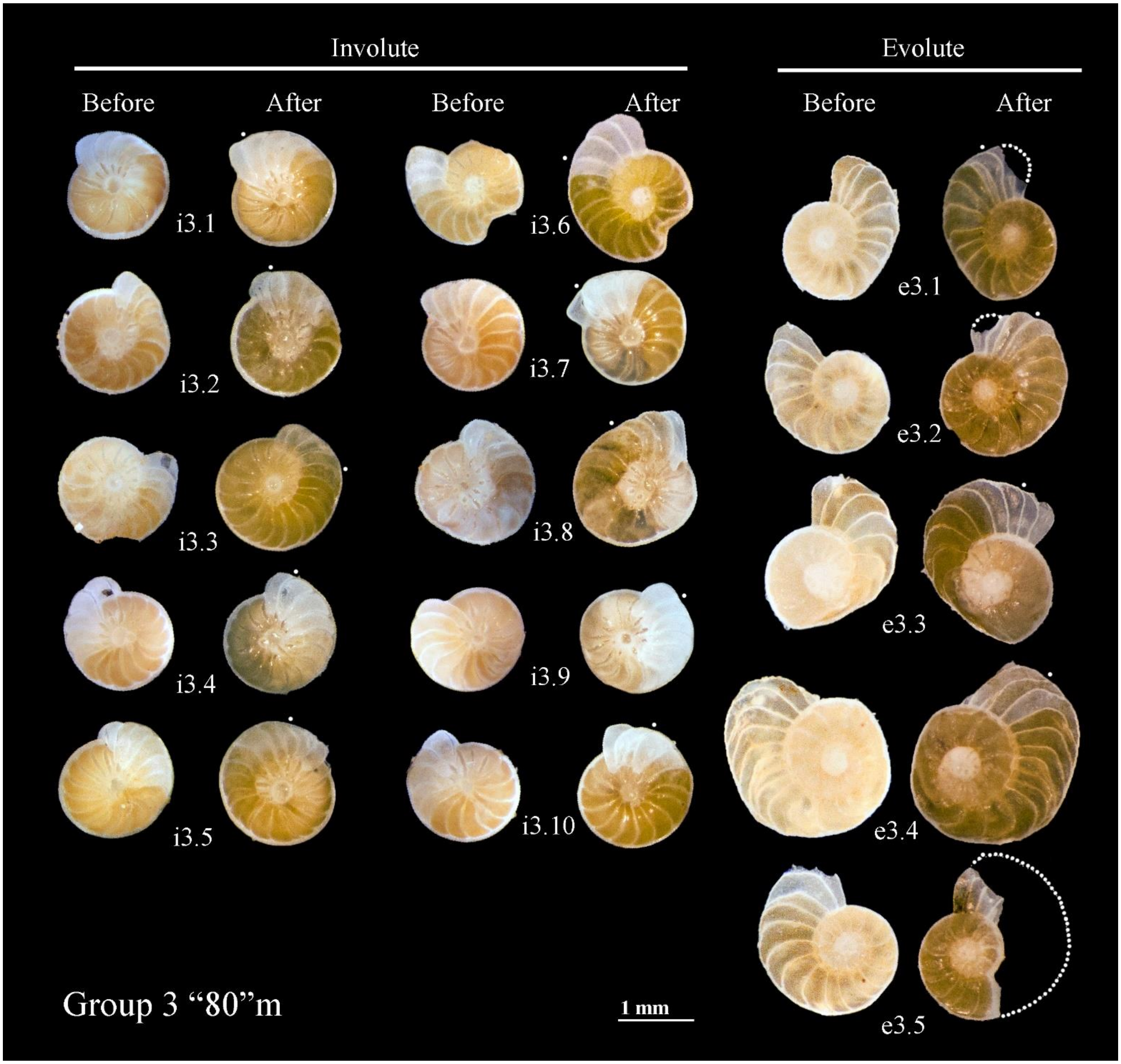

Figure S3: Photographs of all specimens from group 3 before and after the experiment. Labels represent specimen ID. White dots mark the newly formed chambers. Broken specimens outline was estimated for area calculations**.**

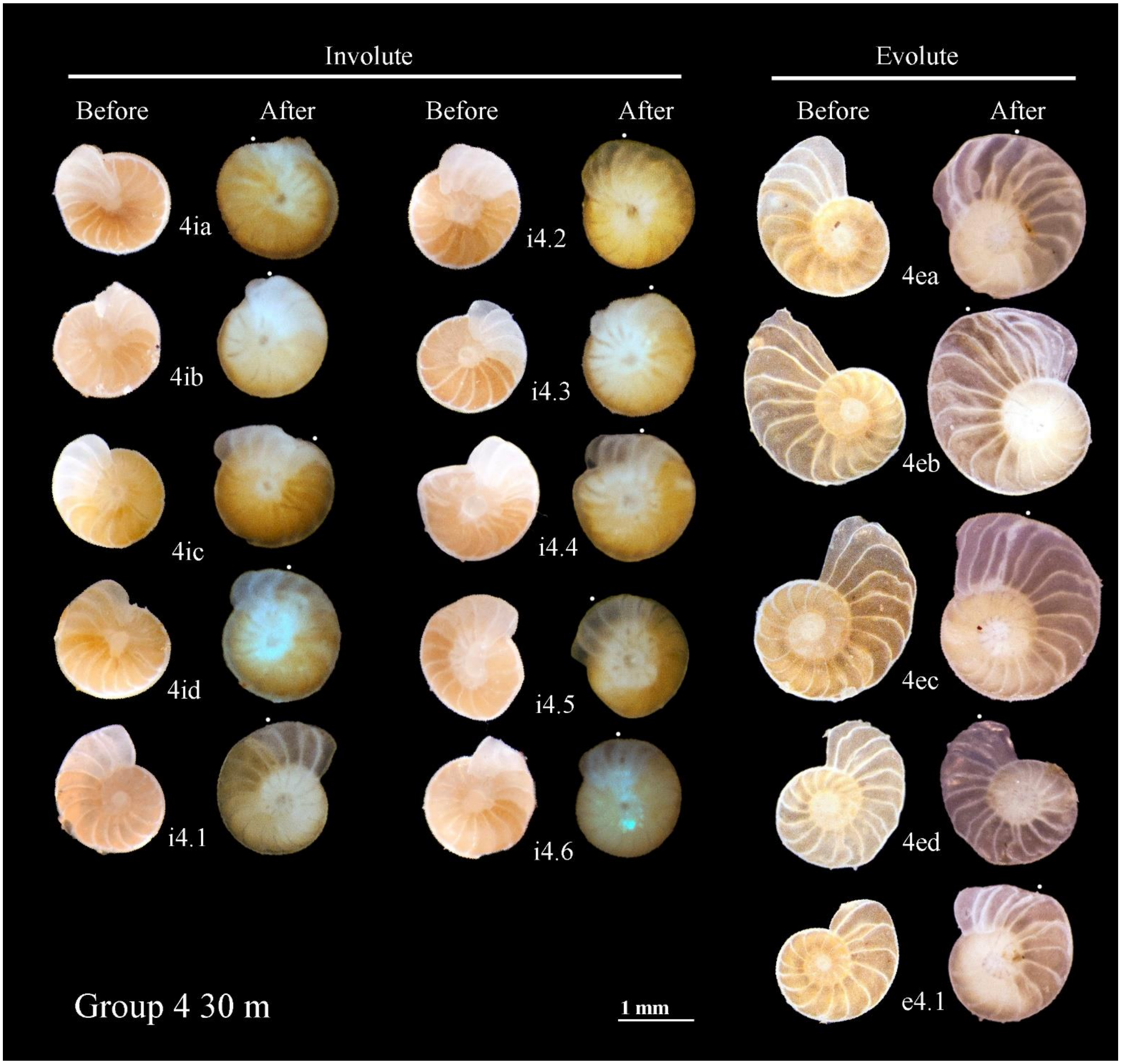

Figure S4: Photographs of all specimens from group 4 before and after the experiment. Labels represent specimen ID. White dots mark the newly formed chambers.

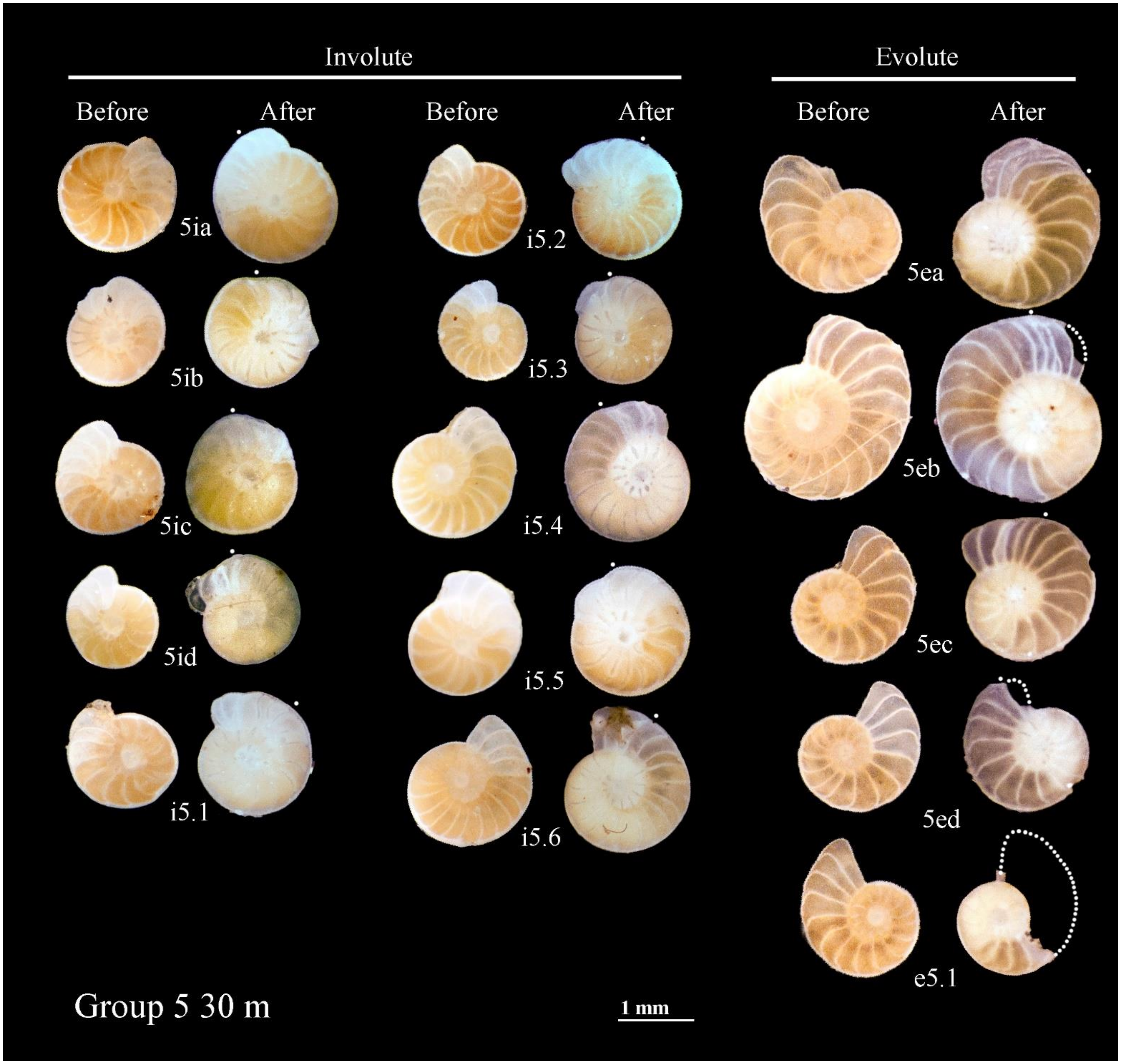

Figure S5: Photographs of all specimens from group 5 before and after the experiment. Labels represent specimen ID. White dots mark the newly formed chambers. Broken specimens outline was estimated for area calculations**.**

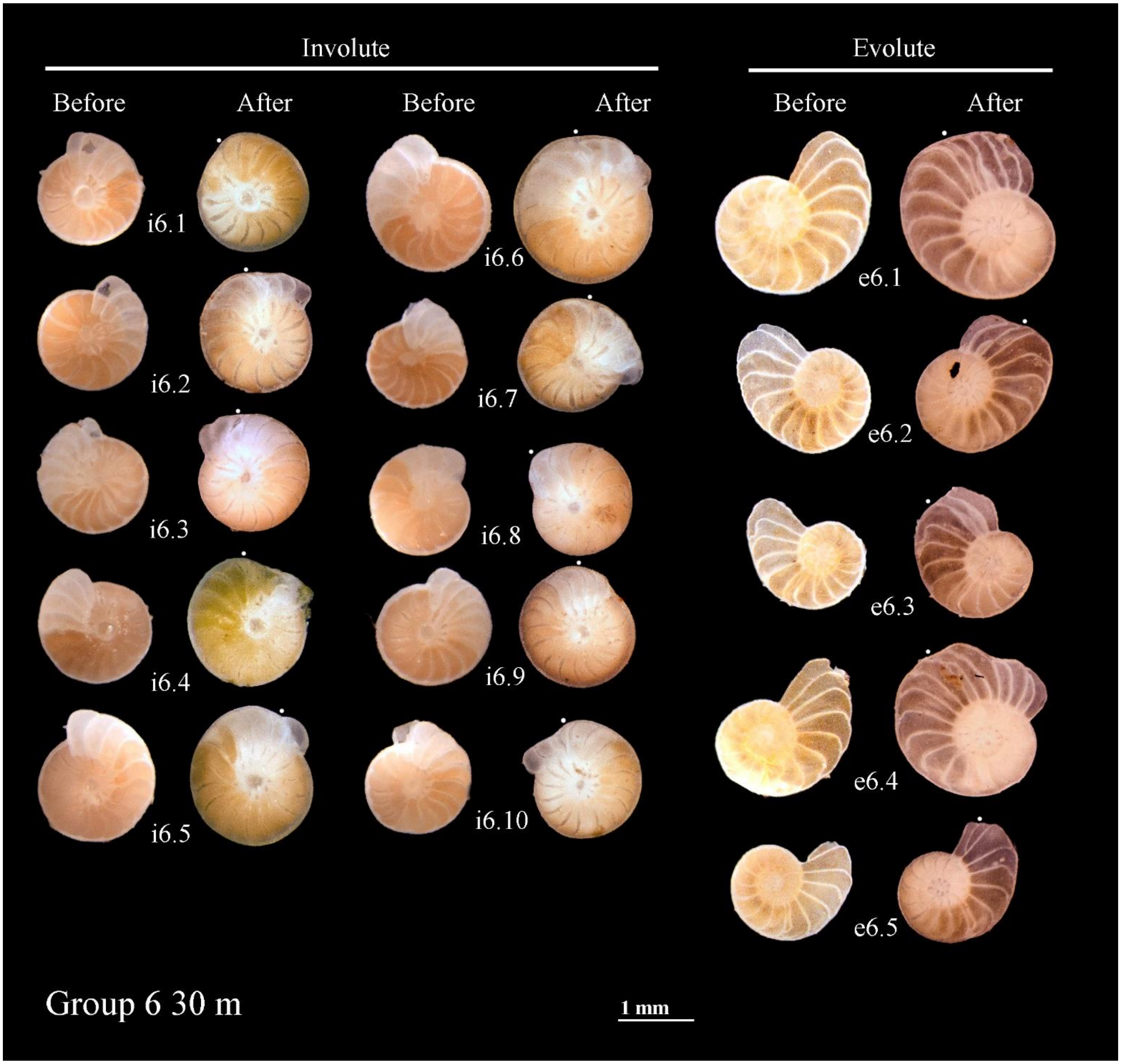

Figure S6: Photographs of all specimens from group 6 before and after the experiment. Labels represent specimen ID. White dots mark the newly formed chambers.

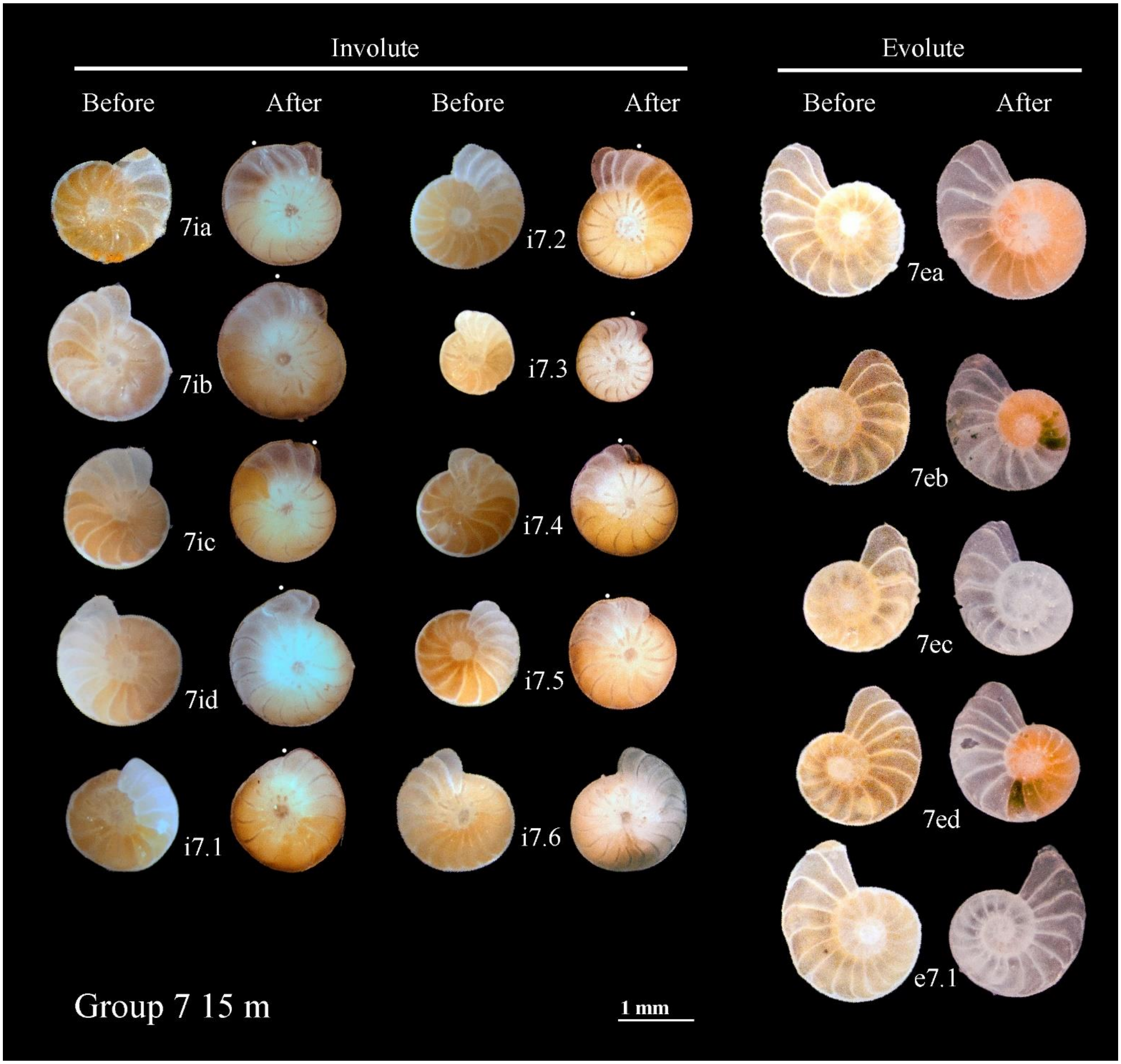

Figure S7: Photographs of all specimens from group 7 before and after the experiment. Labels represent specimen ID. White dots mark the newly formed chambers. All evolute specimens in the "after" photographs are bleached and dead. The orange color visible in some of the specimens is possibly free-living alga inhabiting the empty shells.

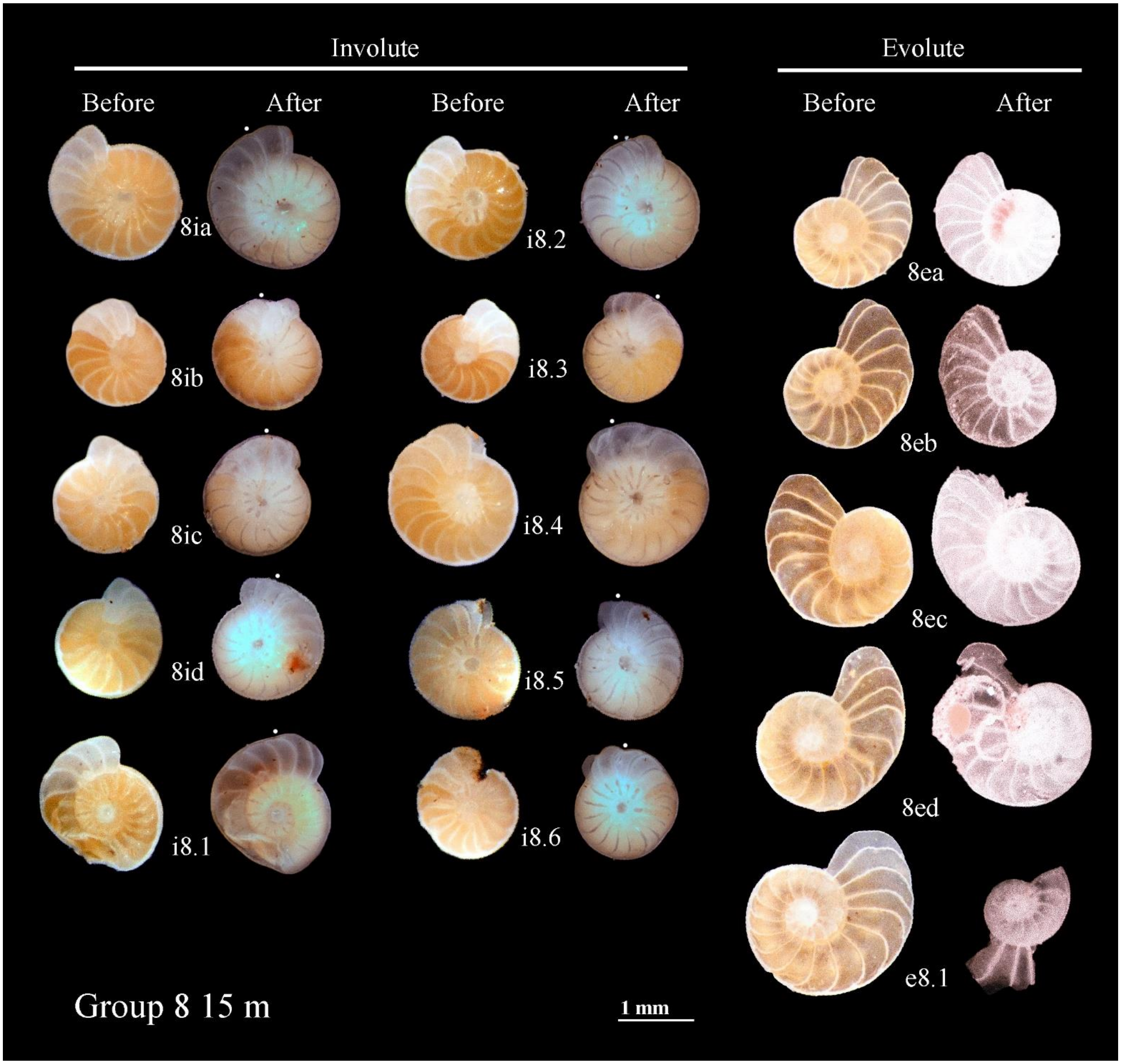

Figure S8: Photographs of all specimens from group 8 before and after the experiment. Labels represent specimen ID. White dots mark the newly formed chambers.

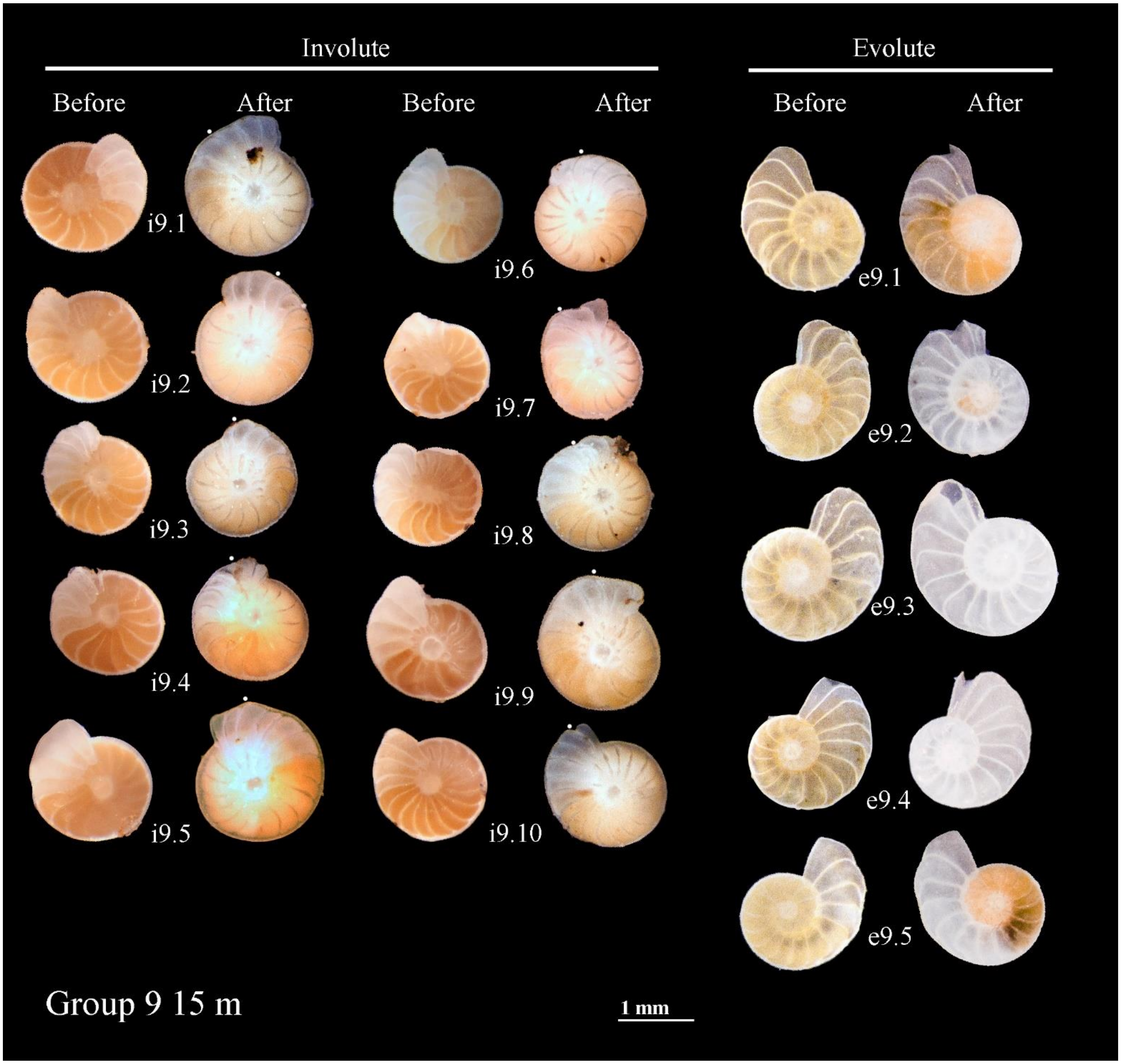

Figure S9: Photographs of all specimens from group 9 before and after the experiment. Labels represent specimen ID. White dots mark the newly formed chambers. All evolute specimens in the "after" photographs are bleached and dead. The orange color visible in some of the specimens is possibly free-living alga inhabiting the empty shells.

Supplementary tables

Table S1: Normality tests. Shapiro-Wilk test for all data sets.

	Area			Weight			Chlorophyll		
	Depth	df (N)	p	Depth	df (N)	p	Depth	df (N)	p
Involute	“80”	10	0.033*	“80”	10	0.250	“80”	10	0.131
	45	20	0.005*	45	18	0.167	45	8	0.323
	30	30	0.210	30	30	0.058	30	8	0.930
	15	30	0.091	15	25	0.194	15	8	0.027*
Evolute	“80”	5	0.719	“80”	4	0.171	“80”	6	0.699
	45	10	0.991	45	9	0.118	45	8	0.713
	30	15	0.373	30	14	0.150	30	8	0.916
	15	15	-**	15	12	-**	15	-**	-**

*Normal distribution ($p<0.05$)
**0% survival (all other groups have 100% survival)

Table S2: Area statistics. Non-parametric Mann–Whitney test comparing added area means.

	Groups	N (total)	p-value
Involute	“80”m_15m	40	0.050*
	“80”m_30m	40	0.148
	“80”m_45m	30	0.880
	30m_45m	50	0.208
	15m_30m	60	0.000*
	15m_45m	50	0.021*
Evolute	“80”m_15m	20	0.000*
	“80”m_30m	20	0.612
	“80”m_45m	15	0.129
	30m_45m	25	0.683
	15m_30m	30	0.000*
	15m_45m	25	0.000*

*Significant difference ($p<0.05$).

Table S3: Weight statistics. Non-parametric Mann–Whitney test comparing added weight means.

	Groups	N (total)	p-value
Involute	“80”m_15m	35	0.001*
	“80”m_30m	40	0.002*
	“80”m_45m	28	1.000
	30m_45m	48	0.001*
	15m_30m	55	0.651
	15m_45m	43	0.001*
Evolute	“80”m_15m	16	0.000*
	“80”m_30m	18	0.061
	“80”m_45m	13	0.330
	30m_45m	23	0.643
	15m_30m	26	0.000*
	15m_45m	21	0.000*

*Significant difference ($p<0.05$).

Table S4: Chlorophyll statistics. Non-parametric Mann–Whitney test comparing chlorophyll means.

	Groups	N (total)	p-value
Involute	“80”m_15m	18	0.001*
	“80”m_30m	18	0.001*
	“80”m_45m	18	0.929
	30m_45m	16	0.001*
	15m_30m	16	0.052
	15m_45m	16	0.001*
Evolute	“80”m_15m	14	0.001*
	“80”m_30m	14	0.002*
	“80”m_45m	14	0.796
	30m_45m	16	0.001*
	15m_30m	16	0.000*
	15m_45m	16	0.000*

*Significant difference ($p<0.05$)
